# Physiologically Based Pharmacokinetic Modeling of Venetoclax in Pediatric Acute Myeloid Leukemia: Food Effects, Age-Related Pharmacokinetics, and Voriconazole-Mediated Drug–Drug Interactions

**DOI:** 10.3390/pharmaceutics18101335

**Published:** 2026-10-21

**Authors:** Yanming Li, Mengmeng Liu, Qixian Ling, Ning Sun, Huyong Zheng, Peng Guo, Libo Zhao

**Affiliations:** 1 Department of Pharmacy, Beijing Children’s Hospital, Capital Medical University, National Center for Children’s Health, Beijing 100045, China; 2 Department of Pharmacy, Peking University Third Hospital, Beijing 100191, China; 3 Department of Leukemia, Beijing Children’s Hospital, Capital Medical University, National Center for Children’s Health, Beijing 100045, China

**Keywords:** venetoclax, pediatric population, acute myeloid leukemia, PBPK modeling, Chinese population

## Abstract

**Background:** Venetoclax (VEN), a selective B-cell lymphoma 2 inhibitor, has been increasingly used off-label in pediatric patients with hematologic malignancies. However, its pharmacokinetic characteristics in pediatric patients remain insufficiently characterized. **Methods:** An adult VEN physiologically based pharmacokinetic (PBPK) model was developed and extrapolated to children using age-dependent physiological and CYP3A4 ontogeny data. The pediatric model was calibrated and evaluated using data from 76 children with AML (203 plasma concentrations) and subsequently applied to assess food effects, age-related pharmacokinetic differences, and VEN dose-reduction scenarios during VCZ co-administration. The clinical study was registered with the Chinese Clinical Trial Registry (ChiCTR1900027146 and ChiCTR2400080307). **Results:** The adult and pediatric models adequately reproduced the observed VEN concentration–time profiles. At 200 mg/m^2^, a normal-fat meal increased predicted AUC_inf_ and C_max_ by 2.74- and 1.77-fold, respectively, versus a low-fat meal. Children aged 2–<6 years had the highest exposure; AUC_inf_ was 1.44- and 1.91-fold higher than in the 6–<12- and 12–<18-year groups, respectively. During VCZ co-administration, reducing VEN to 50% and 25% of the full dose decreased AUC_0–168h_ by 36.8% and 60.5% and C_max_ by 42.5% and 70.5%, respectively, with minimal changes in T_max_. **Conclusions:** The pediatric VEN PBPK model quantitatively characterized the effects of meal conditions, developmental age, and VCZ-mediated CYP3A inhibition on VEN pharmacokinetics. These findings support administration with a normal diet, closer safety monitoring in children aged 2–<6 years receiving 200 mg/m^2^, and model-informed evaluation of VEN dose reduction during concomitant VCZ therapy.

## 1. Introduction

Venetoclax (VEN) is a selective inhibitor of B-cell lymphoma 2 (BCL-2) that promotes tumor cell apoptosis by blocking BCL-2-mediated anti-apoptotic signaling [1]. VEN was initially approved for the treatment of chronic lymphocytic leukemia/small lymphocytic lymphoma in adults and was subsequently approved, in combination with azacitidine, decitabine, or low-dose cytarabine, for newly diagnosed acute myeloid leukemia (AML) in adults aged ≥75 years or in those with comorbidities that preclude the use of intensive induction chemotherapy [2].

VEN is administered orally and has low aqueous solubility, and its systemic exposure is markedly influenced by food intake [3]. Studies in adults have shown that, compared with fasting conditions, administration with a low-fat meal increases VEN exposure by approximately 3.4-fold, whereas administration with a high-fat meal increases exposure by approximately 5.1–5.3-fold [4]. In vitro studies have demonstrated that VEN is predominantly metabolized by CYP3A, making its pharmacokinetics particularly susceptible to CYP3A inhibition [5]. This interaction is especially relevant in patients with AML, who frequently require triazole antifungal agents, such as voriconazole (VCZ), for the prophylaxis or treatment of invasive fungal infections during therapy [6]. As a strong CYP3A inhibitor, VCZ can substantially reduce VEN clearance and increase systemic exposure, necessitating substantial dose reductions in VEN during concomitant administration [7].

The effects of CYP3A inhibition on VEN pharmacokinetics have been relatively well characterized in adults through both clinical and model-based studies [8,9,10,11,12,13]. A previous physiologically based pharmacokinetic (PBPK) analysis predicted that the strong CYP3A inhibitor ketoconazole could increase the maximum plasma concentration (C_max_) of VEN by approximately 2.0–2.5-fold and the area under the concentration–time curve from time 0 to infinity by approximately 5.8–7.8-fold [13]. A study involving nine patients with AML and one patient with myelodysplastic syndrome, showed that, when VEN 100 mg was administered once daily concomitantly with VCZ 200 mg every 12 h, the peak concentration of VEN was comparable to that observed with VEN 400 mg once daily as monotherapy, whereas the trough concentration was markedly increased [11]. In 2025, a population pharmacokinetic (PopPK) modeling study in adults with hematologic malignancies showed that VEN doses of 75–100 mg/day during concomitant VCZ administration might result in higher VEN exposure than VEN 400 mg/day administered alone, whereas an approximately 50 mg/day dose produced an exposure level closer to that achieved with VEN 400 mg/day monotherapy [10].

In recent years, VEN-based chemotherapy regimens have been explored in pediatric patients with AML and have demonstrated promising antileukemic activity and acceptable safety in clinical studies [14,15,16,17,18]. Meanwhile, two PopPK studies have characterized VEN pharmacokinetics in pediatric patients and provided important evidence for pediatric dose optimization. One study incorporated allometric scaling and a CYP3A maturation function to account for developmental changes in body size and CYP3A activity [19], whereas a more recent real-world study in Chinese pediatric patients characterized pharmacokinetic (PK) variability using clinically relevant covariates, including body surface area and concomitant triazole therapy [20]. Although these PopPK studies have substantially advanced the understanding of VEN pharmacokinetics and dose selection in children, the effects of clinically relevant factors such as different dietary conditions and specific CYP3A-mediated drug–drug interactions, particularly concomitant voriconazole administration, have not been systematically evaluated across pediatric developmental stages [21].

Such questions are difficult to address comprehensively through conventional pediatric pharmacokinetic studies because pediatric drug research is constrained by ethical considerations, limited patient populations, restricted blood-sampling volumes, and substantial physiological differences across developmental stages [22]. Physiologically based pharmacokinetic (PBPK) modeling is a widely recognized, effective, and reliable tool that accounts for relevant physiological changes in pediatric patients and helps to overcome many of the limitations inherent in pediatric drug research [23]. By integrating age-dependent physiological characteristics with drug-specific properties, PBPK models enable mechanistic extrapolation from adults to children and allow drug exposure to be simulated under clinical scenarios that are difficult to adequately investigate in clinical trials [24]. This approach is therefore particularly suitable for evaluating age-related pharmacokinetic differences, food effects, and CYP3A-mediated drug–drug interactions, and may provide additional quantitative support for pediatric dose selection and individualized therapy.

Therefore, in the present study, a pediatric VEN PBPK model was first developed based on a validated adult VEN PBPK model in combination with clinical pharmacokinetic data from Chinese pediatric patients with AML. The model was then used to simulate age-related differences in VEN pharmacokinetic characteristics and to investigate the effects of different dietary conditions and concomitant VCZ administration on systemic VEN exposure. In addition, different VEN dose-reduction regimens during concomitant VCZ administration were simulated, with the aim of providing a basis for individualized VEN dosing and dose optimization during co-administration with strong CYP3A inhibitors in pediatric patients with AML.

## 2. Materials and Methods

### 2.1. Workflow and Software

**Figure 1:**
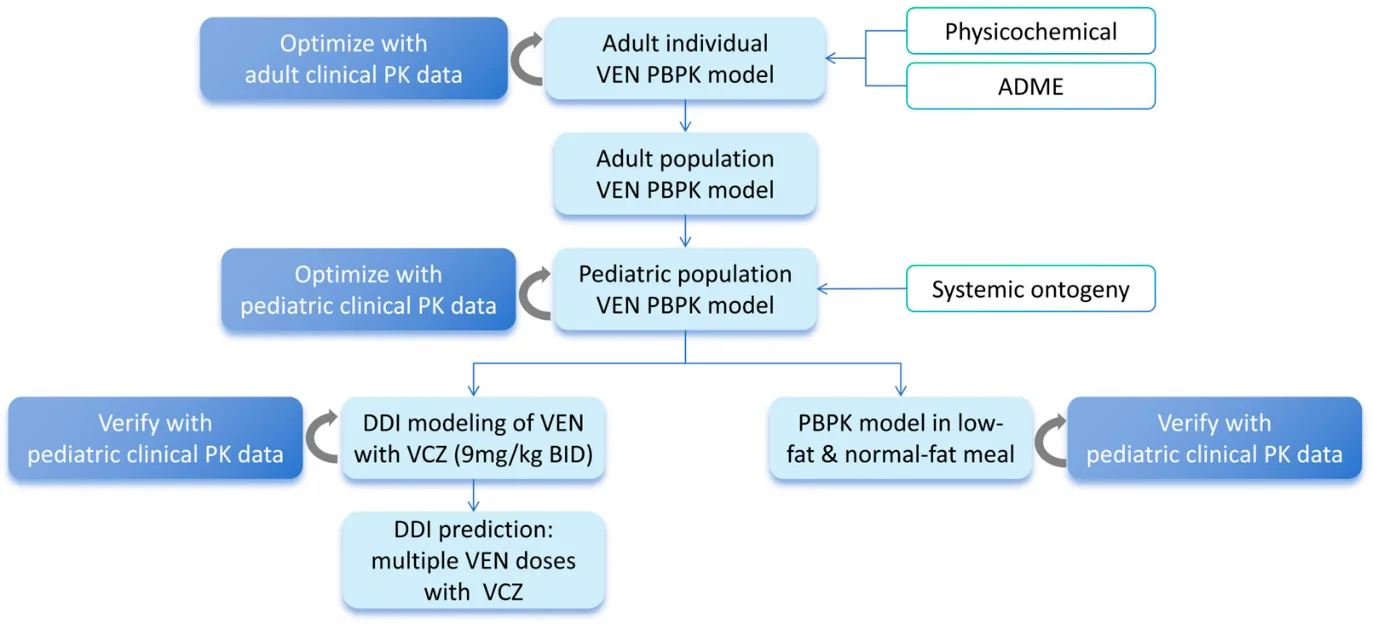
Schematics for the PBPK modelling workflow. VEN—venetoclax; PBPK—physiologically based pharmacokinetic; PK—pharmacokinetics, ADME—absorption, distribution, metabolism, and excretion; DDI—drug–drug interaction; VCZ—voriconazole.

A modeling strategy combining bottom-up and top-down approaches was applied to develop, validate, and perform clinical scenario simulations of the VEN PBPK model using PK-Sim^®^ (version 12.2, part of the Open Systems Pharmacology Suite, https://www.open-systems-pharmacology.org/, accessed on 30 August 2026) [25]. The modeling workflow is shown in Figure 1.

Physicochemical properties and drug-specific parameters were obtained from the DrugBank database (https://go.drugbank.com/drugs/DB11581, accessed on 30 August 2026) and related literature. Clinical PK data of VEN in Chinese adults were identified through searches of the PubMed database and related publications. The details of the search strategy and literature selection process are provided in the Supplementary Materials (Table S4 and Figure S3). For plasma concentration–time profiles available only in graphical format, data points were digitized using GetData Graph Digitizer (version 2.20, S. Fedorov). Clinical PK data of VEN in Chinese pediatric patients with AML were obtained from clinical studies conducted at Beijing Children’s Hospital, Capital Medical University. Venetoclax (VEN; AbbVie Inc., North Chicago, IL, USA) was used in the clinical studies. The study protocol was reviewed and approved by the Ethics Committee of Beijing Children’s Hospital and was conducted in accordance with the Declaration of Helsinki and relevant ethical guidelines. Written informed consent was obtained from the legal guardians of all participants. Pediatric patients with sufficient age and cognitive capacity provided informed assent according to ethical requirements. The studies were registered in the Chinese Clinical Trial Registry (ChiCTR1900027146; ChiCTR2400080307). Data processing, statistical analysis, and graphical visualization were performed using Microsoft Excel, R software (version 4.3.3, R Foundation for Statistical Computing), and RStudio (version 2024.04.0, Posit Software, PBC).

### 2.2. Basic PBPK Model Development

In this study, an adult PBPK model of VEN was developed based on a previously published PBPK model. A virtual individual representing a typical East Asian female was generated using mean physiological parameters. Based on this individual, a virtual population consisting of 100 healthy adults was generated. The virtual population included 50 males and 50 females aged 18–81 years.

The initial adult VEN PBPK model was developed by integrating drug-specific physicochemical properties and in vitro absorption, distribution, metabolism, and excretion (ADME) parameters (Table S1) [25]. As CYP3A4 is the major enzyme responsible for VEN metabolism, CYP3A4-mediated metabolism was initially assumed to account for VEN metabolic clearance during model development. Based on the clinical plasma concentration data obtained from healthy volunteers following a single oral dose of VEN 100 mg under low-fat meal conditions (Table S2), the model was calibrated by parameter identification using a Monte Carlo approach combined with sensitivity analysis to estimate the model parameters.

Key PK parameters were derived from the model simulations. Model performance was evaluated by visual comparison of the simulated plasma concentration–time profiles with the corresponding observed clinical data. Predictive performance was further assessed using goodness-of-fit (GOF) plots comparing model-predicted and observed plasma concentrations.

### 2.3. Extrapolation to Pediatric PBPK Model

The validated adult VEN PBPK model was subsequently extrapolated to the pediatric population while retaining the drug-specific physicochemical and ADME parameters established in the adult model. Age-dependent anatomical, physiological, and enzyme ontogeny information was incorporated to account for developmental changes in pediatric patients. The resulting pediatric model was further calibrated and evaluated using clinical PK data from pediatric patients with AML.

Age-dependent physiological and anatomical characteristics, including body height, body weight, organ volumes, and organ blood flows, were generated using the built-in pediatric population algorithms implemented in PK-Sim. The ontogeny of CYP3A4, the major enzyme involved in VEN metabolism, was incorporated using the age-dependent enzyme expression profiles available within the PK-Sim physiological database. Other age-dependent physiological processes relevant to drug disposition, including plasma protein binding and renal function, were also accounted for using the corresponding pediatric physiological information implemented in PK-Sim.

Clinical PK data collected from pediatric patients with AML at Beijing Children’s Hospital were used for model calibration. Parameters identified as influential on VEN exposure were optimized within physiologically plausible ranges. Model performance within the calibration dataset was subsequently assessed by comparing the simulated concentration–time profiles with the observed pediatric data.

To investigate the effect of dietary conditions on VEN pharmacokinetics in children, two dietary scenarios representing low-fat and normal-fat meals were incorporated into the pediatric PBPK model. Drug-specific disposition parameters were kept consistent across dietary conditions.

The goodness of fit of the calibrated pediatric PBPK model was assessed at both the individual and population levels. Simulated concentration–time profiles were compared with the observed concentrations, and GOF plots were used to assess agreement between model-predicted and observed values. At the population level, model performance was further examined by determining whether the observed concentrations were adequately captured within the model-predicted 95% prediction interval.

To further characterize age-related differences in VEN pharmacokinetics, virtual pediatric populations were generated for three age groups: 2–<6, 6–<12, and 12–<18 years. Each age group comprised 100 virtual individuals, with equal numbers of males and females. Age-stratified simulations were performed following a single oral VEN dose of 200 mg/m^2^ under low-fat meal conditions.

### 2.4. Development and Evaluation of the Venetoclax–Voriconazole DDI PBPK Model

To evaluate the effect of VCZ-mediated drug–drug interaction (DDI) on VEN exposure in pediatric patients with AML, a DDI PBPK model was developed based on the established pediatric VEN PBPK model. VCZ was incorporated into the simulation at a dose of 9 mg/kg twice daily (BID), together with its disposition processes and inhibitory effect on CYP3A4.

The integrated VEN–VCZ DDI model was evaluated using clinical VEN concentration data from pediatric patients receiving concomitant VEN and VCZ therapy. Model performance was assessed at both the individual and population levels by comparing model-predicted VEN concentration–time profiles with the corresponding observed concentrations. At the population level, the agreement between observed concentrations and the model-predicted profiles and 95% prediction intervals was visually assessed.

Following model evaluation, VEN exposure was simulated under different dose-reduction scenarios during continued VCZ co-administration under low-fat dietary conditions. Three VEN dose levels were evaluated: the full clinical dose of 200 mg/m^2^, 50% of the full dose (100 mg/m^2^), and 25% of the full dose (50 mg/m^2^). The effects of dose reduction were compared using AUC_0–168h_ and C_max_, while the VCZ dosing regimen and other simulation conditions were kept unchanged across scenarios.

## 3. Results

### 3.1. Development and Evaluation of the Adult and Pediatric VEN PBPK Models

**Figure 2:**
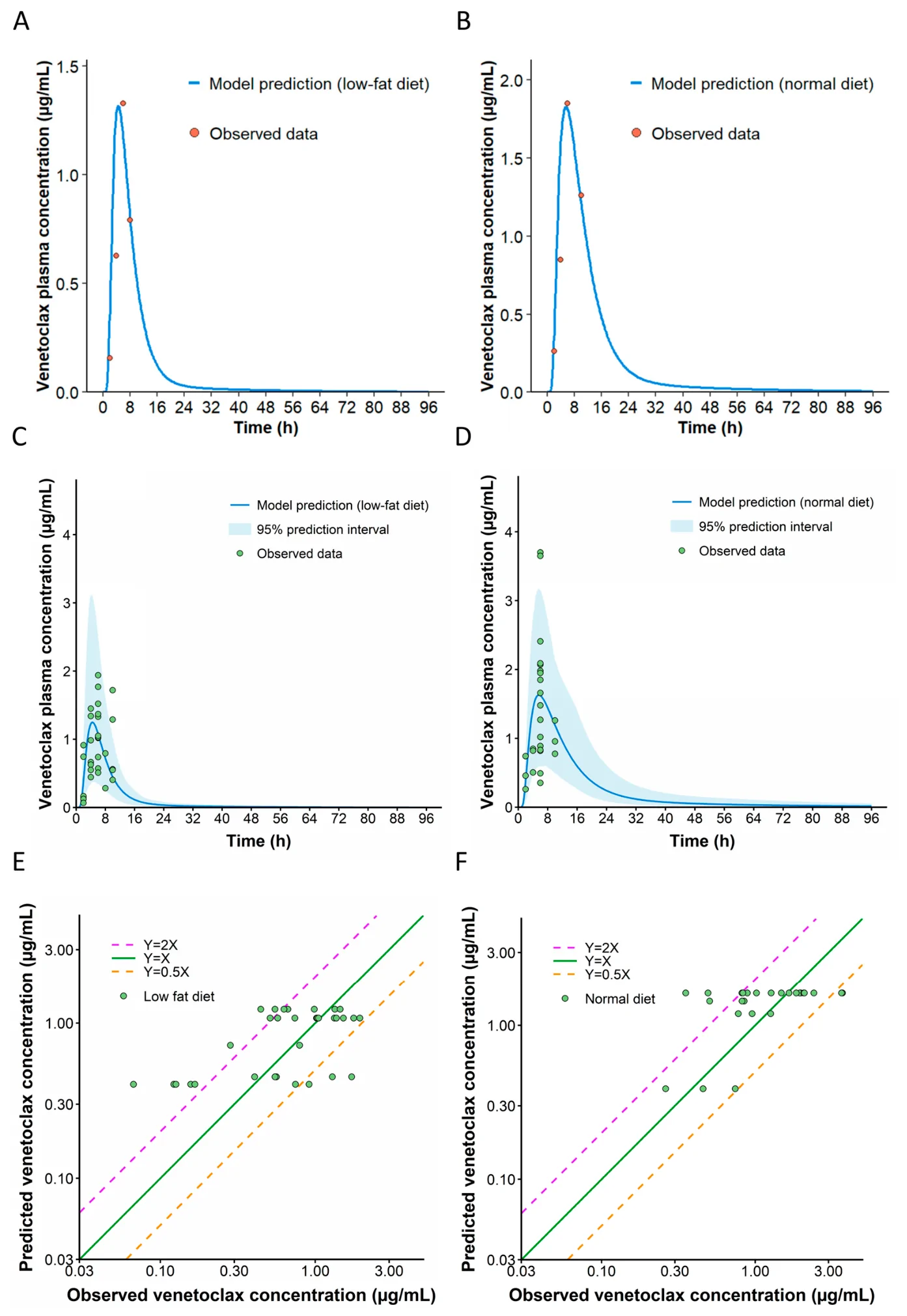
Development and validation of the pediatric PBPK model for venetoclax under low-fat and normal dietary conditions. (**A**,**B**) Individual fits of the pediatric PBPK model under low-fat (**A**) and normal diet (**B**) conditions. The line represents the model-simulated VEN concentration–time profile, and dots represent observed data. (**C**,**D**) Population fits of the pediatric PBPK model for plasma VEN concentrations under low-fat (**C**) and normal diet (**D**) conditions. The line represents the model-predicted concentration–time profile, the shaded area indicates the 95% prediction interval, and dots represent observed data. (**E**,**F**) Goodness-of-fit plots comparing predicted versus observed VEN concentrations under low-fat (**E**) and normal diet (**F**) conditions. Both axes are displayed on logarithmic scales. The solid line represents the line of identity (y = x), and the dashed lines indicate twofold deviation (y = 0.5x and y = 2x). Symbols represent the observed data under the corresponding dietary conditions.

An adult VEN PBPK model was successfully developed and validated using published clinical pharmacokinetic data. Model performance was assessed by simulating plasma concentration–time profiles following a single 100-mg oral dose of VEN administered to healthy Chinese Han subjects after a low-fat breakfast. The predicted plasma concentration–time profiles showed good agreement with the observed data. GOF plots further demonstrated overall concordance between predicted and observed plasma concentrations across the evaluated single-dose scenarios. Individual model fits, population model fits, and GOF plots of the adult model are presented in Figure S1, whereas the evaluation results of the pediatric PBPK model are shown in Figure 2.

The adult PBPK model was subsequently extrapolated to the pediatric population. Model parameters were further optimized using clinical PK data from pediatric patients with AML, and the performance of the calibrated pediatric model was assessed by comparing model predictions with the observed concentration data. Population simulations were performed in 100 virtual Asian pediatric subjects to establish the pediatric PBPK model. Model performance was evaluated by comparing the simulated concentration–time profiles with observed clinical PK data from Chinese pediatric AML patients receiving VEN. A total of 76 pediatric patients were included, contributing 203 plasma concentration observations following oral administration of VEN. The median age was 9.45 years (range, 0.92–15.87 years), and the median body weight was 27.0 kg (range, 8.6–122.0 kg). Of the 76 patients, 45 (59.2%) were male and 31 (40.8%) were female. Detailed demographic and baseline characteristics are summarized in Table S3.

### 3.2. PBPK Model Predictions Under Different Dietary Conditions and Across Pediatric Age Groups

The established pediatric VEN PBPK model was used to evaluate the effects of dietary conditions on VEN pharmacokinetics. Plasma concentration–time profiles were simulated following a single oral dose of VEN at 200 mg/m^2^ under low-fat (400 kcal) and normal diet (600 kcal) conditions. Compared with the low-fat diet, the normal diet substantially increased VEN exposure, with the predicted mean AUC_inf_ increasing from 11.55 ± 7.60 to 31.64 ± 16.95 μmol·h/L, corresponding to an approximately 2.74-fold increase. The mean C_max_ also increased from 1.46 ± 1.05 to 2.59 ± 1.31 μmol/L, representing an approximately 1.77-fold increase. In addition, T_max_ was delayed from 4.57 ± 0.49 to 5.45 ± 0.78 h, while the terminal half-life increased from 20.75 ± 4.98 to 26.57 ± 2.48 h. The predicted pharmacokinetic parameters under different dietary conditions are summarized in Table 1. The simulated population concentration–time profiles under both dietary conditions were generally consistent with the observed clinical concentrations, with most observations falling within the corresponding 95% prediction intervals (Figure 2).

Subsequently, age-stratified simulations were performed in three pediatric subgroups: 2–<6, 6–<12, and 12–<18 years (Figure S2). Age-dependent differences in VEN pharmacokinetics were observed across the three age groups (Table 1). Under the 200 mg/m^2^ dosing regimen, VEN exposure was highest in children aged 2–<6 years, as reflected by higher AUC_inf_ and C_max_ values. The mean AUC_inf_ in the 2–<6-year group was 12.24 ± 6.92 μmol·h/L, approximately 1.44- and 1.91-fold higher than that in children aged 6–<12 years (8.49 ± 4.33 μmol·h/L) and 12–<18 years (6.40 ± 2.82 μmol·h/L), respectively. Similarly, the mean C_max_ was 1.58 ± 0.93 μmol/L in children aged 2–<6 years, approximately 1.50- and 2.16-fold higher than that in the 6–<12-year (1.05 ± 0.57 μmol/L) and 12–<18-year (0.73 ± 0.32 μmol/L) groups, respectively. V_ss_/F was also highest in the youngest age group (14.04 ± 12.76 L/kg), approximately 1.46- and 1.80-fold higher than that in the 6–<12-year and 12–<18-year groups, respectively. CL/F decreased progressively with age, from 1.32 ± 1.05 L/h/kg in children aged 2–<6 years to 0.91 ± 0.59 and 0.66 ± 0.44 L/h/kg in the older age groups. The terminal half-life increased with age, from 17.39 ± 2.18 h in children aged 2–<6 years to 21.72 ± 1.90 h in those aged 6–<12 years and 26.57 ± 1.89 h in those aged 12–<18 years. T_max_ remained relatively stable across age groups, ranging from 4.49 to 4.58 h.

### 3.3. DDI Model Evaluation and Dose Adjustment

**Figure 3:**
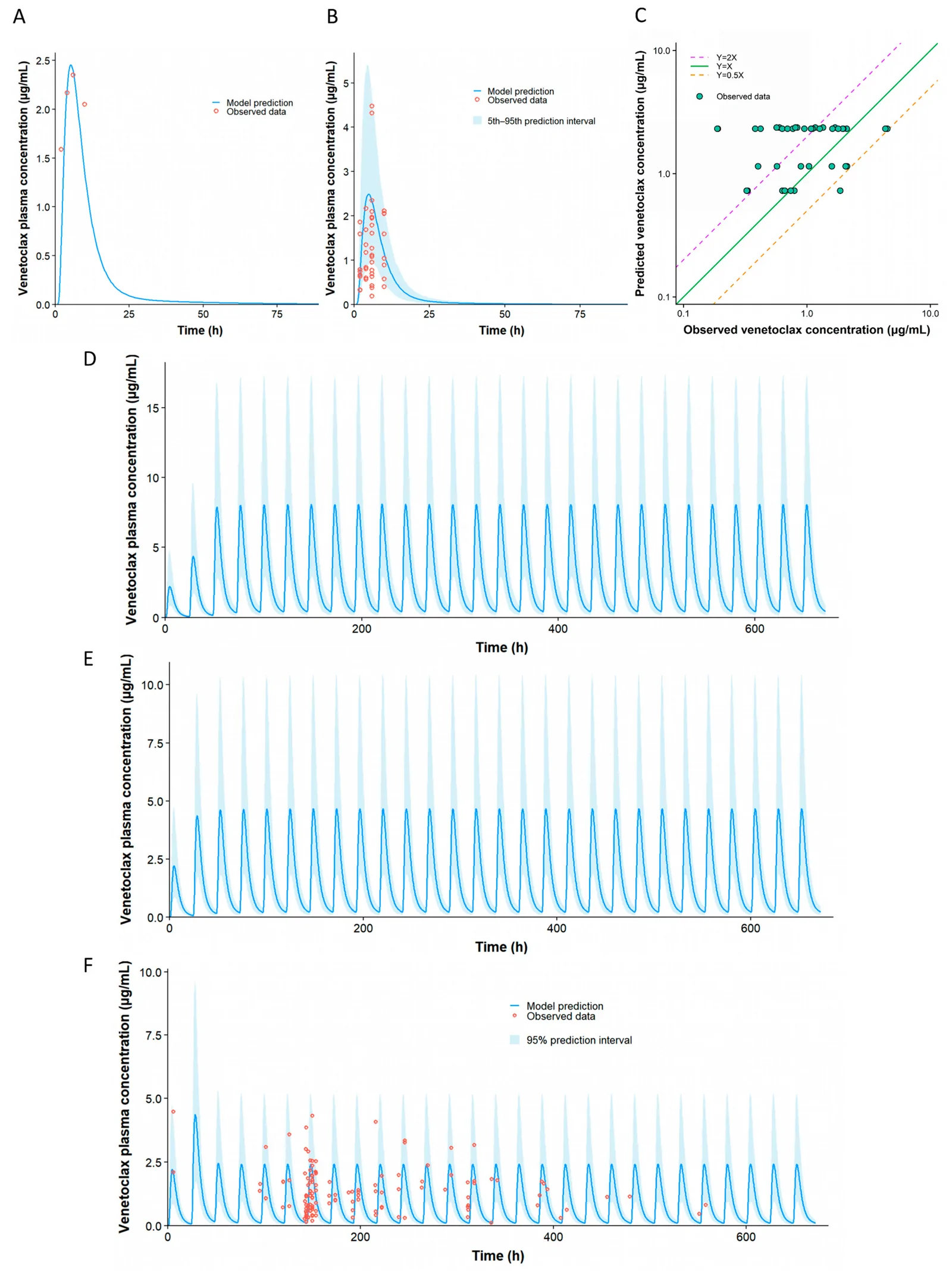
Physiologically based pharmacokinetic model evaluation and simulation of venetoclax under voriconazole-mediated CYP3A inhibition in pediatric patients. (**A**) Individual model fit of venetoclax pharmacokinetics in a representative pediatric patient receiving concomitant voriconazole. The solid line represents the model-predicted concentration–time profile, and red symbols represent observed plasma concentrations. (**B**) Population model fit of venetoclax in pediatric patients under voriconazole co-administration. The solid line indicates the population-predicted profile, the shaded area represents the 95% prediction interval, and red symbols denote observed concentrations. (**C**) Goodness-of-fit plot comparing population-predicted and observed venetoclax concentrations under voriconazole co-administration. The solid line represents the line of identity (y = x), and the dashed lines represent the two-fold boundaries (y = 2x and y = 0.5x). (**D**,**E**) Simulated steady-state venetoclax concentration–time profiles during voriconazole co-administration under different venetoclax dose-adjustment scenarios, representing the full-dose regimen (**D**) and the reduced-dose regimen (**E**). (**F**) Validation of the pediatric VEN–VCZ PBPK model using clinical data. The solid line represents the model-predicted concentration–time profile, the shaded area indicates the prediction interval, and red symbols represent observed plasma concentrations.

The established VEN–VCZ DDI PBPK model adequately characterized VEN pharmacokinetics in pediatric patients receiving concomitant VCZ therapy. As shown in Figure 3A, in a representative individual, the model-predicted VEN concentration–time profile was in good agreement with the observed concentrations, indicating that the model adequately captured individual VEN pharmacokinetic characteristics during VCZ co-administration. At the population level, most observed concentrations were within the model-predicted 95% prediction interval, further supporting the predictive performance of the DDI model (Figure 3B). Model performance was further assessed using a goodness-of-fit plot comparing predicted and observed VEN concentrations, with most predictions falling within the 0.5- to 2-fold bounds, although overprediction was evident for some lower observed concentrations (Figure 3C). As shown in Figure 3F, the predicted VEN concentration–time profiles were generally consistent with the observed concentrations, with most measured values falling within the 95% prediction interval.

Based on the evaluated DDI model, VEN exposure was subsequently simulated under different dose-adjustment scenarios during VCZ co-administration under low-fat dietary conditions. As summarized in Table 2, the predicted AUC_0–168h_ was 459.11 μmol·h/L with the full VEN dose and decreased to 290.12 μmol·h/L and 181.44 μmol·h/L when the VEN dose was reduced to 50% and 25% of the full dose, respectively. Compared with the full-dose regimen, these reductions corresponded to decreases in cumulative VEN exposure of approximately 36.8% and 60.5%, respectively. A similar dose-dependent pattern was observed for C_max_, which decreased from 9.51 ± 5.75 μmol/L with the full dose to 5.47 ± 3.27 μmol/L with the 50% dose and 2.81 ± 1.66 μmol/L with the 25% dose, representing reductions of approximately 42.5% and 70.5%, respectively. T_max_ showed minimal variation across the three regimens. Overall, progressive VEN dose reduction substantially reduced systemic exposure during VCZ co-administration, with the 25% dose producing the lowest cumulative exposure and peak concentration among the evaluated regimens (Figure 3D–F).

## 4. Discussion

In the present study, a PBPK model of VEN was established by extrapolating a verified adult model and incorporating age-dependent physiological characteristics and clinical pharmacokinetic (PK) data from Chinese pediatric patients with AML. The calibrated pediatric model adequately characterized the observed VEN concentration–time profiles and showed satisfactory agreement between predicted and observed concentrations. It was subsequently applied to evaluate three clinically relevant determinants of VEN exposure: dietary conditions, developmental age, and concomitant VCZ administration.

Dietary conditions substantially influenced VEN exposure in the pediatric simulations. Compared with the low-fat condition, normal-fat meals produced an approximately twofold increase in AUC, accompanied by higher C_max_ and relatively modest changes in T_max_. This direction is consistent with adult clinical data, although the magnitude should not be compared directly because our simulations compared different fed states rather than fasting versus fed conditions. In healthy adults, a low-fat meal increased VEN C_max_ and AUC by approximately 3.4-fold relative to fasting, whereas a high-fat meal further increased both parameters by approximately 50% relative to a low-fat meal [26,27]. Pooled PopPK analyses likewise estimated increases in VEN bioavailability of 2.99-, 3.77-, and 4.25-fold after low-, moderate-, and high-fat meals, respectively, compared with fasting [26].

Because VEN is highly lipophilic and poorly soluble, gastrointestinal conditions are expected to play an important role in determining its oral absorption. The mechanistic PBPK analysis reported by Emami Riedmaier et al. showed that the VEN food effect could be described by incorporating biorelevant solubility, dissolution, and intestinal absorption processes [4]. These findings suggest that food-induced changes in bile secretion, micellar solubilization, dissolution, gastric emptying, and intestinal conditions may collectively modify VEN absorption [28]. The modest shift in the time to peak concentration observed in our simulations further suggests that dietary conditions may affect not only the extent of absorption but also its temporal pattern.

The food effect may be particularly relevant in pediatric AML because dietary conditions can fluctuate considerably during intensive treatment. Nausea, vomiting, oral mucositis, infection, loss of appetite, and nutritional interventions are common challenges in pediatric oncology and may lead to substantial variation in caloric intake and meal composition [28]. Therefore, dietary status may represent an additional, and potentially modifiable, source of interindividual variability in pediatric VEN therapy. Thus, maintaining reasonably consistent administration conditions and documenting meal intake may help reduce an avoidable source of exposure variability during pediatric VEN therapy.

Age-stratified simulations further demonstrated substantial developmental differences in VEN pharmacokinetics under the empirically used 200 mg/m^2^ regimen in our Chinese pediatric AML setting. Children aged 2–<6 years had the highest weight-normalized CL/F and a larger V_ss_/F, yet also showed the highest AUC and C_max_, with AUC approximately 1.45- and 1.93-fold higher than that in the 6–<12- and 12–<18-year groups, respectively. The coexistence of higher weight-normalized clearance and higher exposure is not necessarily contradictory: with BSA-based dosing, smaller children receive a relatively greater dose on an mg/kg basis, which may partly offset their higher CL/F per kilogram [29]. More broadly, drug absorption, tissue distribution, plasma protein binding, liver size, hepatic blood flow, and metabolic capacity undergo nonparallel developmental changes throughout childhood [30,31], indicating that the observed age-related patterns cannot be attributed to clearance or body size alone.

These findings are consistent with emerging pediatric VEN PopPK evidence. Badawi et al. identified body weight as a significant determinant of both clearance and volume of distribution and incorporated allometric scaling together with CYP3A maturation when deriving pediatric VEN doses [19]. More recently, Zhao et al. reported typical CL/F and V/F in Chinese pediatric patients and identified BSA and concomitant triazole use as significant determinants of CL/F, whereas total protein influenced V/F [20]. These absolute PopPK estimates cannot be directly compared with our weight-normalized CL/F and V_ss_/F because of differences in model structure, parameter definition, and normalization; nevertheless, both studies support the multifactorial nature of VEN disposition in children. Taken together, the age-related exposure differences observed in our simulations suggest that whether the empirically used 200 mg/m^2^ regimen achieves appropriate exposure across developmental stages warrants confirmation in prospective pediatric PK and exposure–response studies.

A major application of the present model was the evaluation of VEN dose adjustment during concomitant VCZ therapy. Previous clinical studies with ketoconazole and posaconazole, as well as PBPK analyses of CYP3A inhibition, have consistently demonstrated that inhibition of this pathway markedly reduces VEN clearance and increases systemic exposure [13,32]. In the present simulations, progressive dose reduction during VCZ co-administration decreased both AUC_0–168h_ and C_max,_ whereas T_max_ remained essentially unchanged. Notably, the reduction in cumulative exposure was less than proportional to the reduction in dose, indicating that considerable VEN exposure may persist despite substantial dose reduction under CYP3A inhibition. This pattern is consistent with adult clinical data. Gao et al. reported that VEN 100 mg/day combined with VCZ produced a C_max_ comparable to that of VEN 400 mg/day alone, whereas the trough concentration remained substantially higher, suggesting that peak concentration alone may not adequately reflect persistent exposure during the interaction [11]. Similarly, Yang et al. found that VEN 75 and 100 mg/day with VCZ produced greater AUC than VEN 400 mg/day alone, whereas 50 mg/day yielded an AUC closer to the adult reference; VEN exposure also continued to accumulate over two weeks, with a median day-14/day-7 AUC24 accumulation ratio of approximately 1.2 [10]. These observations support consideration of cumulative exposure, rather than C_max_ alone, when evaluating VEN dose reduction during VCZ co-administration.

Importantly, our previous clinical experience in Chinese pediatric AML provides direct pediatric evidence supporting this interaction [17]. Wen et al. treated 45 children with newly diagnosed AML using an empirical VEN regimen of 200 mg/m^2^/day, with the dose reduced to 25% during co-administration with strong CYP3A inhibitors and to 50% with moderate inhibitors. Despite these dose adjustments, children receiving azoles had significantly higher VEN trough concentrations than those not receiving azoles (1711.1 vs. 504.4 ng/mL), whereas peak concentrations did not differ significantly between the groups. This clinical observation is consistent with the persistence of VEN exposure predicted in the present dose-reduction simulations. The magnitude of the VEN–VCZ interaction may nevertheless vary substantially among individuals. In a dedicated PBPK analysis, Dong et al. predicted that VCZ could increase VEN exposure by 4.5–9.6-fold and demonstrated that the magnitude of the interaction was influenced by CYP2C19 phenotype through its effect on VCZ pharmacokinetics [9]. CYP2C19 polymorphism is a major source of variability in VCZ exposure, with substantially greater VCZ exposure predicted in poor metabolizers, thereby altering the degree of CYP3A4 inhibition experienced by VEN. However, CYP2C19 genotyping was not routinely performed in our pediatric cohort, and genotype-specific VCZ pharmacokinetic data were therefore unavailable. Consequently, the present simulations represent population-level predictions and could not be reliably stratified into CYP2C19 poor, intermediate, and normal metabolizer phenotypes. Consistent with this interindividual variability, age-stratified VPC and GOF analyses showed no clear age-dependent systematic bias, although some deviations between predicted and observed concentrations remained around the peak-concentration region, which may partly reflect interindividual variability in venetoclax absorption and the magnitude of the voriconazole-mediated interaction (Figures S4 and S5). These findings help to explain why a uniform VEN dose reduction may not result in comparable exposure across patients and why adult fixed-dose recommendations cannot be directly translated to a BSA-based pediatric regimen.

Accordingly, the present dose-scenario analysis should be interpreted as a quantitative comparison of exposure reduction rather than identification of an optimal pediatric VEN dose. Among the regimens evaluated, the 25% dose produced the lowest AUC_0–168h_ and C_max_; however, because a well-validated VEN therapeutic exposure range has not yet been established specifically for pediatric AML, these findings cannot establish that this regimen provides optimal exposure or the best efficacy–toxicity balance. Measurement of VEN concentrations, ideally together with VCZ exposure and, where clinically relevant, CYP2C19 genotype, may therefore help to inform individualized management of this DDI [9,10]. Prospective pediatric studies integrating VEN and VCZ pharmacokinetics with efficacy and toxicity outcomes are needed to determine the most appropriate dose-adjustment strategy during concomitant VCZ therapy.

Several limitations should be acknowledged. First, children younger than 2 years were not included in the age-stratified scenario simulations because of the limited clinical PK data available for this age group and rapid developmental changes in physiology and CYP3A maturation introduce greater uncertainty into model extrapolation [31]. Second, the current model primarily focused on CYP3A4-mediated VEN metabolism and did not explicitly characterize all minor disposition pathways or other sources of interindividual variability.

## 5. Conclusions

In conclusion, the developed pediatric PBPK model adequately characterized VEN pharmacokinetics in children with AML and identified dietary conditions, developmental age, and concomitant VCZ therapy as important determinants of VEN exposure. At a VEN dose of 200 mg/m^2^, administration with a normal meal increased AUCinf and Cmax by approximately 2.74- and 1.77-fold, respectively, compared with a low-fat meal, supporting administration with a normal diet rather than intentional restriction to a low-fat diet to facilitate adequate oral absorption. Under the 200 mg/m^2^ dosing regimen, children aged 2–<6 years exhibited higher systemic exposure and a larger apparent distribution volume than older children, suggesting that closer monitoring for treatment-related adverse effects may be warranted in this age group. During concomitant VCZ therapy, reducing the VEN dose to 50% and 25% of the full dose decreased AUC0–168h by 36.8% and 60.5% and Cmax by 42.5% and 70.5%, respectively. Collectively, these findings provide a quantitative basis for model-informed optimization of VEN therapy in pediatric AML, with particular consideration of meal conditions, age-related pharmacokinetic differences under the current dosing regimen, and dose adjustment during concomitant VCZ treatment.

## Figures and Tables

**Table 1:** Predicted Venetoclax Pharmacokinetic Parameters in Different Pediatric Simulation Scenarios Following a 200 mg/m^2^ Dose.

Scenario	CL/F (L/h/kg)	V_ss_/F (L/kg)	T_max_ (h)	t_1/2_ (h)	AUC_inf_ (μmol·h/L)	C_max_ (μmol/L)
Dietary condition						
Low-fat diet	-	-	4.57 ± 0.49	20.75 ± 4.98	11.55 ± 7.60	1.46 ± 1.05
Normal diet	-	-	5.45 ± 0.78	26.57 ± 2.48	31.64 ± 16.95	2.59 ± 1.31
Age Group (years)						
2–<6	1.32 ± 1.05	14.04 ± 12.76	4.58 ± 0.47	17.39 ± 2.18	12.24 ± 6.92	1.58 ± 0.93
6–<12	0.91 ± 0.59	9.62 ± 6.91	4.49 ± 0.45	21.72 ± 1.90	8.49 ± 4.33	1.05 ± 0.57
12–<18	0.66 ± 0.44	7.81 ± 5.36	4.54 ± 0.43	26.57 ± 1.89	6.40 ± 2.82	0.73 ± 0.32

PK parameters are represented as mean ± standard deviation (SD).

**Table 2:** Predicted Venetoclax Pharmacokinetic Parameters under Different Dose-Adjustment Scenarios during Voriconazole Coadministration under Low-Fat Conditions.

Dose Adjustment	T_max_ (h)	AUC_0–168h_ (μmol·h/L)	C_max_ (μmol/L)
Full VEN dose	4.87 ± 0.61	459.11	9.51 ± 5.75
50% VEN dose	4.69 ± 0.53	290.12	5.47 ± 3.27
25% VEN dose	4.63 ± 0.50	181.44	2.81 ± 1.66

PK parameters are presented as mean ± standard deviation (SD), except for AUC0–168 h, which was calculated from the population arithmetic mean concentration–time profile using the linear trapezoidal method.

## Data Availability

The data can be shared up on request.

## Supplementary Materials

The following supporting information can be downloaded at: https://www.mdpi.com/article/10.3390/pharmaceutics18101335/s1, Figure S1: Development and validation of the PBPK model for VEN in adults; Figure S2: Age-stratified visual predictive checks in pediatric patients; Figure S3: Literature search and study selection process for venetoclax PBPK model validation; Figure S4: Age-stratified visual predictive check of venetoclax concentrations during concomitant voriconazole administration in pediatric AML patients; Figure S5: Age-stratified goodness-of-fit analyses of venetoclax concentrations during concomitant voriconazole administration in pediatric AML patients; Table S1: Input parameters for developing the venetoclax PBPK model; Table S2: The clinical study used for developing and validating the PBPK model; Table S3: Patient demographics and baseline characteristics; Table S4: Optimized PBPK model parameters and parameter estimation precision values; Table S5: Search strategy for published venetoclax pharmacokinetic data.

## Abbreviations

The following abbreviations are used in this manuscript:

AML	Acute myeloid leukemia
AUC_0–168h_	Area Under the plasma concentration–time curve from time 0 to 168 h
AUC_inf_	Area under the concentration–time curve
ChiCTR	Chinese Clinical Trial Registry
CL	Clearance
CL/F	Apparent clearance (clearance/bioavailability)
C_max_	Maximum concentration
DDI	Drug–drug interactions
GOF	Goodness of fit
PBPK	Physiologically based pharmacokinetic
PK	Pharmacokinetic
PopPK	Population pharmacokinetic
T_max_	Time to maximum concentration
VCZ	Voriconazole
VEN	Venetoclax
V_ss_/F	Apparent volume of distribution at steady state
