# Supplementary material for: Physiologically Based Pharmacokinetic Modeling of Venetoclax in Pediatric Acute Myeloid Leukemia: Food Effects, Age-Related Pharmacokinetics, and Voriconazole-Mediated Drug–Drug Interactions
Source: Pharmaceutics. 2026 Oct 21;18(10):1335. doi: 10.3390/pharmaceutics18101335 (PMC13708470; doi:10.3390/pharmaceutics18101335)
Supplement: Supplementary file 1 [file pharmaceutics-18-01335-s001.zip › pharmaceutics-4565138-supplementary.pdf]

Table S1. Input parameters for developing the venetoclax PBPK model.

Parameter	Initial value	Reference	Final value	Source
**Physiochemical properties/formulation**				
Log P	8	[1]	5.22	Optimized
Molecular Weight (g/mol)	868.44	[1]	868.44	-
Compound type	Ampholyte	[1]	Ampholyte	-
Solubility (μg/mL)	4.5 (pH 7.4)	[1]	4.5 (pH 7.4)	-
pKa	3.4(acid), 10.3(base)	[1]	3.4(acid), 10.3(base)	-
Dissolution time (50% dissolved) (min)	60	[2]	60	-
Dissolution shape	1.2	[2]	1.2	-
Lag time (min)	30	[2]	30	-
**Absorption**				
Specific intestinal permeability (cm/s)	1.8×10^{-5}	[2]	1.8×10^{-5}	-
**Distribution**				
f_u	0.0001	[1]	0.0001	-
Blood/Plasma concentration ratio	0.574	[1]	0.574	-
Partition coefficients	Rodgers and Rowland method	Default	Rodgers and Rowland method	-
Cellular permeabilities	Charge-dependent Schimitt normalized to PK-Sim	Default0	Charge-dependent Schimitt normalized to PK-Sim	-
**Metabolism**				
CYP3A4 CL_{int} (μL/min/pmol rec. enzyme)	26	[2]	26	-
Non-CYP3A4 clearance (min^{-1})	86	[2]	Not parameterized	-

[1] Emami Riedmaier A, Lindley DJ, Hall JA, Castleberry S, Slade RT, Stuart P, et al. Mechanistic physiologically based pharmacokinetic modeling of the dissolution and food effect of a biopharmaceutics classification system iv compound-the venetoclax story. J Pharm Sci. 2018;107(1):495–502.

[2] Development of Physiology Based Pharmacokinetic Model to Predict the Drug Interactions of Voriconazole and Venetoclax

Table S2. The clinical study used for developing and validating the PBPK model.

Reference	Zhendong Chen et al. 2021	Pediatric clinical cohort from CCLG-AML
Year	2021	2024
Ethnicity	Chinese	Chinese
Participants, n	6	76
Population	Healthy	Pediatric AML
Age (years)	26.4 (3.06)	9.45 (0.92–15.87)
Treatment	100 mg single dose	200 mg/m^2 qd po
Model role	Validation	Validation
Dietary status	Fasting	Low-fat or normal-fat meal; administered within 30 min after a meal
Sampling schedule	Serial plasma sampling after a single VEN dose, as reported in the original study	Pre-dose samples on Days 7 and 14, with optional samples at 2, 4, 6, and 10 h post-dose on the same days.
Concomitant treatment	None	With or without voriconazole

Table S3. Patient demographics and baseline characteristics.

**Characteristic**	**Pediatric AML**
Number of patients	76
Number of observations	203
Sex, No. (%)	
Male	45 (59.2)
Female	31 (40.8)
Age, years, No. (%)	
Median (range)	9.45 (0.92–15.87)
0 to ＜1	1 (1.3)
1 to ＜2	10 (13.2)
2 to ＜10	32 (42.1)
10 - 18	33 (43.4)
Baseline body weight, kg	
Mean (SD)	33.2 (21.9)
Median (range)	27.0 (8.6–122.0)
Risk stratification, No. (%)	
Standard/Intermediate risk	26 (34.2)
High risk	50 (65.8)
Therapy, No. (%)	
CCLG-AML 2019 supplementary study—venetoclax therapy protocol	60 (78.9)
CCLG-AML 2024 protocol	16 (21.1)

Table S4. Optimized PBPK model parameters and parameter estimation precision values.

Parameter	Initial value	Search range	Estimate	%CV	95% CI
Log P	8.0	2-10	5.22	12%	3.99–6.45

Table S5. Search strategy for published venetoclax pharmacokinetic data.

Search concept	Controlled vocabulary / MeSH terms	Free-text terms searched in Title/Abstract	Combination
Pharmacokinetics / drug-concentration monitoring	"Pharmacokinetics"[MeSH Terms] OR "Drug Monitoring"[MeSH Terms]	pharmacokinetic* OR "drug concentration*" OR "plasma concentration*" OR "blood concentration*" OR "serum concentration*" OR "therapeutic drug monitoring" OR TDM OR exposure OR AUC OR Cmax OR Cmin OR trough	#1
Venetoclax	"Venetoclax"[Supplementary Concept]	venetoclax OR "ABT-199" OR "GDC-0199"	#2
Hematologic malignancies	"Hematologic Neoplasms"[MeSH Terms] OR "Leukemia"[MeSH Terms] OR "Leukemia, Myeloid, Acute"[MeSH Terms]	"hematologic malignancy" OR "hematologic malignancies" OR "hematological malignancy" OR "hematological malignancies" OR leukemia OR leukaemia OR "acute myeloid leukemia" OR "acute myeloid leukaemia" OR AML	#3
Final search	—	—	#1 AND #2 AND #3

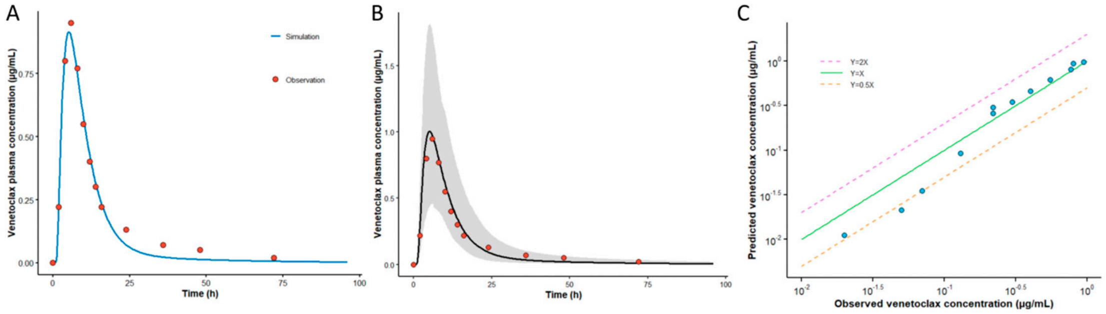

Figure S1. Development and validation of the PBPK model for VEN in adults. (A) Individual fit of the adult PBPK model. The line represents model-simulated VEN concentrations and dots represent observed data. **(B) Population fit of the adult PBPK model for plasma VEN concentrations.** The line represents the model-predicted profile, the shaded area indicates the 95% prediction interval, and dots represent observed data from the published study. **(C) Predicted versus observed VEN concentrations in adults.** The solid line represents the line of identity (y = x), and the dashed lines indicate twofold deviation (y = 0.5x and y = 2x). Symbols denote data from different studies, with most observations distributed within the twofold error range.

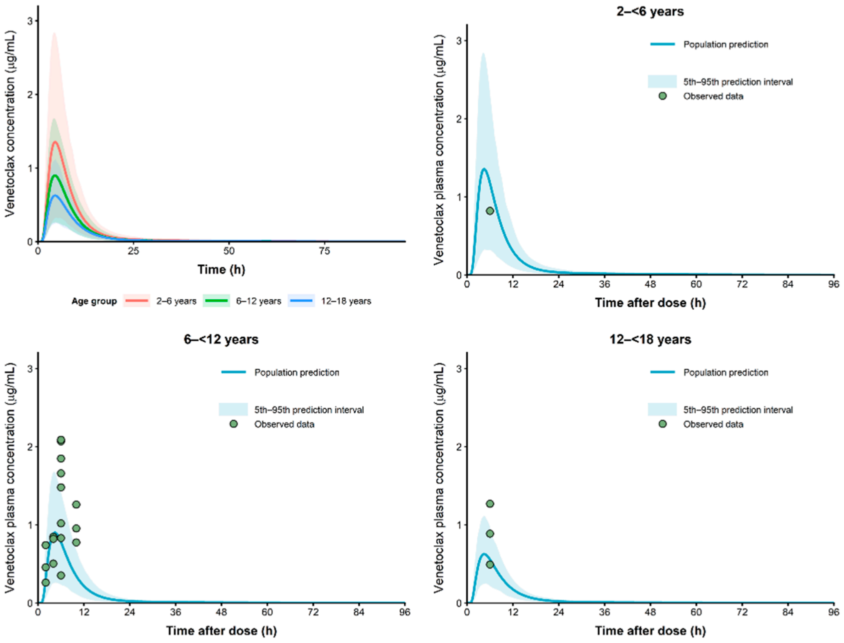

Figure S2. Age-stratified visual predictive checks in pediatric patients.

(A) Simulated population concentration–time profiles of venetoclax across three pediatric age groups (2–<6, 6–<12, and 12–<18 years).

(B–D) Age-stratified visual predictive checks comparing observed venetoclax plasma concentrations with population-predicted concentration–time profiles in patients aged 2–<6 years (B), 6–<12 years (C), and 12–<18 years (D). Solid lines represent population predictions, shaded areas represent the 5th–95th prediction intervals, and symbols represent observed plasma concentrations.

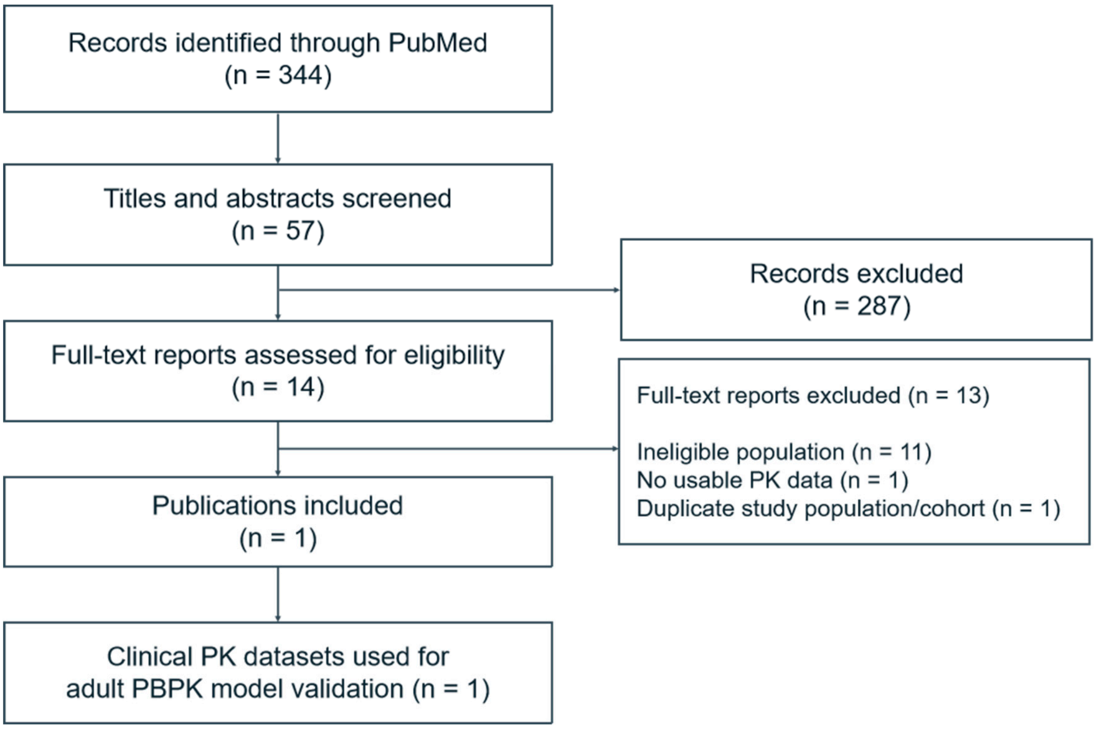

Figure S3. Literature search and study selection process for venetoclax PBPK model validation.

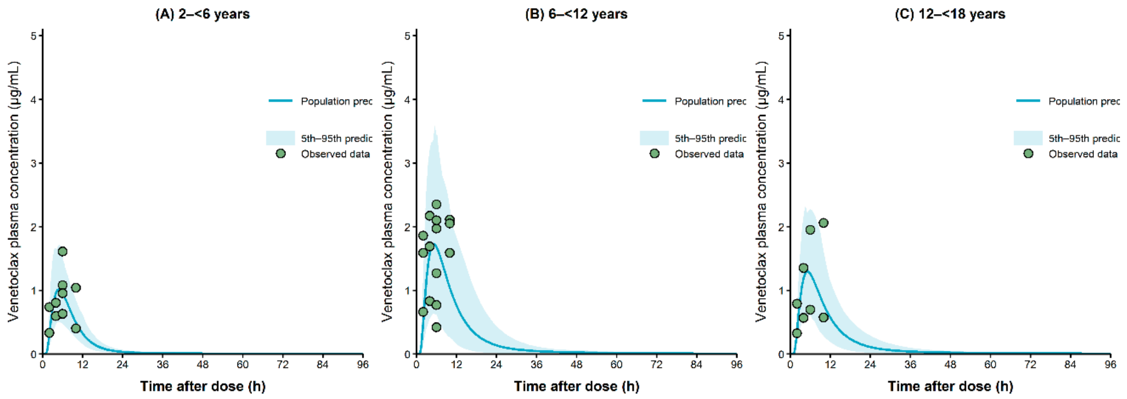

Figure S4. Age-stratified visual predictive check of venetoclax concentrations during concomitant voriconazole administration in pediatric AML patients.

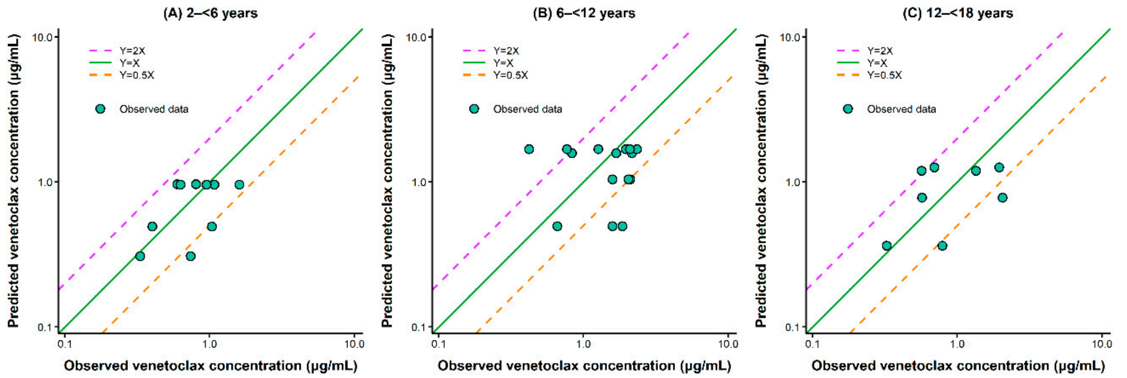

Figure S5. Age-stratified goodness-of-fit analyses of venetoclax concentrations during concomitant voriconazole administration in pediatric AML patients.
